# Supplementary material for: Community composition of aquatic fungi across the thawing Arctic
Source: Sci Data. 2021 Aug 19;8:221. doi: 10.1038/s41597-021-01005-7 (PMC8377128; doi:10.1038/s41597-021-01005-7)
Supplement: Supplementary file 3 — Supplementary Table S2 [file 41597_2021_1005_MOESM3_ESM.pdf]

Supplementary Table S2. Scheme of the three pools of samples and the number of PCR cycles used to create the sequencing libraries.

Pool 1

A - 1	**T1 0.2 31c**	A - 2	**SAS2C 5 28c**	A - 3	**R10 0.2 28c**	A - 4	**GR3 5 28c**	A - 5	**P1 sed 21c**	A - 6		A - 7	**T2 d 28c**	A - 8	**T9 sed 22c**	A - 9	**GR3 sed 21c**	A - 10	**P10 5 20c**	A - 11	**P2 d 22c**	A - 12	
B - 1	**T3 0.2 28c**	B - 2	**B4 5 28c**	B - 3	**R3 5 28c**	B - 4	**GR6 5 28c**	B - 5	**P2 sed 20c**	B - 6		B - 7	**T4 d 28c**	B - 8	**T10 sed 22c**	B - 9	**GR4 sed 21c**	B - 10	**P11 5 21c**	B - 11	**P4 d 25c**	B - 12	
C - 1	**T4 0.2 28c**	C - 2	**P9 0.2 28c**	C - 3	**R7 5 23c**	C - 4	**GR8 5 28c**	C - 5	**P3 sed 23c**	C - 6		C - 7	**T7 d 28c**	C - 8	**T11 sed 25c**	C - 9	**GR5 sed 23c**	C - 10	**6 sed 21c**	C - 11	**P5 d 23c**	C - 12	
D - 1	**T9 0.2 35c**	D - 2	**P9 5 29c**	D - 3	**GR1 0.2 28c**	D - 4	**P1 0.2 24c**	D - 5	**P9 sed 23c**	D - 6		D - 7	**T8 d 28c**	D - 8	**T12 sed 25c**	D - 9	**GR6 sed 23c**	D - 10	**9 pond C2 sed 22c**	D - 11	**P6 d 25c**	D - 12	
E - 1	**T1 5 31c**	E - 2	**P12 5 28c**	E - 3	**GR2 0.2 28c**	E - 4	**P4 0.2 27c**	E - 5	**R3 sed 20c**	E - 6		E - 7	**GR1 d 28c**	E - 8	**B4 0.2 29c**	E - 9	**I1 5 25c**	E - 10	**10 pond B1 sed 22c**	E - 11		E - 12	
F - 1	**T2 5 28c**	F - 2	**R8 0.2 8c**	F - 3	**GR3 0.2 28c**	F - 4	**P8 0.2 27c**	F - 5	**R5 sed 20c**	F - 6		F - 7	**GR2 d 28c**	F - 8	**C2 0.2 27c**	F - 9	**I3 5 25c**	F - 10	**11 sed 22c**	F - 11		F - 12	
G - 1	**T3 5 27c**	G - 2	**R4 0.2 28c**	G - 3	**GR4 0.2 28c**	G - 4	**R4 5 25c**	G - 5	**R6 sed 23c**	G - 6		G - 7	**GR5 d 28c**	G - 8	**C5 0.2 29c**	G - 9		G - 10	neg	G - 11		G - 12	
H - 1	**T5 5 24c**	H - 2	**R7 0.2 28c**	H - 3	**GR2 5 31c**	H - 4	**R2 5 24c**	H - 5	**R7 sed 23c**	H - 6		H - 7	**GR6 d 28c**	H - 8	**I1 0.2 25c**	H - 9		H - 10		H - 11		H - 12	

Pool 2

A - 1	**T2 0.2 28c**	A - 2	**T4 sed 27c**	A - 3	**SAS2A 5 26c**	A - 4	**P11 0.2 28c**	A - 5	**R1 5 26c**	A - 6	**12 pond G1 sed 20c**	A - 7	**GR9 5 30c**	A - 8	**P7 5 24c**	A - 9		A - 10	**T3 d 31c**	A - 11	**T1 d 31c**	A - 12	
B - 1	**T5 0.2 28c**	B - 2	**T5 sed 25c**	B - 3	**SAS2B 5 25c**	B - 4	**P1 5 25c**	B - 5	**R6 5 21c**	B - 6	**14 sed 21c**	B - 7	**GR10 5 30c**	B - 8	**R10 sed 25c**	B - 9		B - 10	**T5 d 28c**	B - 11	**GR3 d 31c**	B - 12	
C - 1	**T6 0.2 28c**	C - 2	**T7 sed 27c**	C - 3	**SAS2D 5 25c**	C - 4	**P3 5 25c**	C - 5	**R8 5 22c**	C - 6	**19 sed 22c**	C - 7	**GR11 5 31c**	C - 8		C - 9	neg	C - 10	**T6 d 25c**	C - 11		C - 12	
D - 1	**T7 0.2 31c**	D - 2	**T8 sed 27c**	D - 3	**G1 5 28c**	D - 4	**P8 5 27c**	D - 5	**R11 5 25c**	D - 6	**P10 sed 21c**	D - 7	**GR12 5 32c**	D - 8		D - 9		D - 10	**P1 d 25c**	D - 11		D - 12	
E - 1	**T4 5 25c**	E - 2	**SAS2A 0.2 31c**	E - 3	**1 sed 28c**	E - 4	**R1 0.2 28c**	E - 5	GR11 0.2 Failed*	E - 6	**P11 sed 22c**	E - 7	**GR12 sed 21c**	E - 8		E - 9		E - 10	**P3 d 25c**	E - 11		E - 12	
F - 1	**T7 5 29c**	F - 2	**SAS2B 0.2 28c**	F - 3	**P2 0.2 27c**	F - 4	**R3 0.2 25c**	F - 5	**GR9 0.2 31c**	F - 6	**R8 sed 21c**	F - 7	**GR7 sed 22c**	F - 8		F - 9		F - 10	**P7 d 26c**	F - 11		F - 12	
G - 1	**T8 5 28c**	G - 2	**SAS2C 0.2 31c**	G - 3	**P3 0.2 29c**	G - 4	**R9 0.2 25c**	G - 5	**GR10 0.2 28c**	G - 6	R11 sed Failed	G - 7	**GR8 sed 22c**	G - 8		G - 9	**P12 d 25c**	G - 10	GR4 d Failed	G - 11		G - 12	
H - 1	**P4 sed 28c**	H - 2	**SAS2D 0.2 31c**	H - 3	**P5 0.2 28c**	H - 4	**R2 0.2 29c**	H - 5	GR12 0.2 Failed	H - 6	**R12 sed 21c**	H - 7	**GR9 sed 22c**	H - 8		H - 9	**P12 sed 25c**	H - 10	GR10 d Failed	H - 11	**GR12 d 30c**	H - 12	

Pool 3

A - 1	**T6 5 22c**	A - 2	**C5 5 23c**	A - 3	**P5 5 26c**	A - 4	**R5 5 22c**	A - 5	**GR11 sed 22c**	A - 6	**T10 0.2 28c**	A - 7	**P2 5 20c**	A - 8		A - 9	**P12 0.2 31c**	A - 10		A - 11	**T9 d 28c**	A - 12	**GR7 d 30c**
B - 1	**T1 sed 25c**	B - 2	**H1 5 232c**	B - 3	**P6 5 23c**	B - 4	**R12 5 22c**	B - 5	**GR5 0.2 28c**	B - 6	**T11 0.2 28c**	B - 7	**P4 5 22c**	B - 8		B - 9	T8 0.2 Failed	B - 10		B - 11	**T10 d 28c**	B - 12	GR8 d Failed
C - 1	**T2 sed 22c**	C - 2	**8 pond C5 sed 23c**	C - 3	**P5 sed 25c**	C - 4	**R1 sed 25c**	C - 5	**GR6 0.2 28c**	C - 6	**T12 0.2 31c**	C - 7	**P10 0.2 22c**	C - 8	**G1 0.2 27c**	C - 9	neg	C - 10		C - 11	**T11 d 28c**	C - 12	**GR9 d 28c**
D - 1	**T3 sed 25c**	D - 2	**16 sed 25c**	D - 3	**P6 sed 28c**	D - 4	**R2 sed 25c**	D - 5	**GR7 0.2 25c**	D - 6	**T9 5 28c**	D - 7	**GR10 sed 20c**	D - 8	**H1 0.2 25c**	D - 9		D - 10		D - 11		D - 12	GR11 d Failed
E - 1	**T6 sed 26c**	E - 2	**17 sed 25c**	E - 3	**P7 sed 25c**	E - 4	**R4 sed 25c**	E - 5	**GR8 0.2 25c**	E - 6	**T10 5 28c**	E - 7	**GR1 5 31c**	E - 8		E - 9		E - 10		E - 11	**P8 d 22c**	E - 12	
F - 1	**B1 0.2 26c**	F - 2	**21 "tiny" sed 25c**	F - 3	**P8 sed 25c**	F - 4	**R9 sed 25c**	F - 5	**R5 0.2 25c**	F - 6	**T11 5 25c**	F - 7	**GR4 5 32c**	F - 8		F - 9		F - 10		F - 11	**P9 d 24c**	F - 12	
G - 1	**B1 5 23c**	G - 2	**P6 0.2 27c**	G - 3	**R9 5 29c**	G - 4	**GR1 sed 25c**	G - 5	**R6 0.2 28c**	G - 6	R11 0.2 Failed	G - 7	**GR5 5 31c**	G - 8		G - 9		G - 10		G - 11	**P10 d 24c**	G - 12	
H - 1	**C2 5 23c**	H - 2	**P7 0.2 27c**	H - 3	**R10 5 22c**	H - 4	**GR2 sed 25c**	H - 5	**I3 0.2 27c**	H - 6	**R12 0.2 29c**	H - 7	**GR7 5 32c**	H - 8		H - 9		H - 10		H - 11	**P11 d 24c**	H - 12	

*samples that did not amplify and were left out of the pools for sequencing
