# Supplementary material for: Community composition of aquatic fungi across the thawing Arctic
Source: Sci Data. 2021 Aug 19;8:221. doi: 10.1038/s41597-021-01005-7 (PMC8377128; doi:10.1038/s41597-021-01005-7)
Supplement: Supplementary file 2 — Supplementary Figure S2 [file 41597_2021_1005_MOESM2_ESM.pdf]

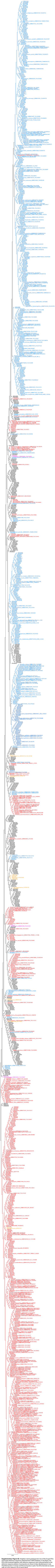

Supplementary Figure S2. Neighbor-Joining phylogenetic tree of the fungal OTUs and fungal references sequences obtained from UNITE database. In the figure, the OTUs that clustered together with reference sequences were coloured based on the reference sequence phylum: Ascomycetes are shown in blue, Basidiomyces in red, Chytridiomycetes in orange and Mucoromycetes in pink. The OTUs that did not cluster with any close reference are in shown black.
